# Everything, altogether, all at once: Addressing data challenges when measuring speech intelligibility through entropy scores

**DOI:** 10.3758/s13428-024-02457-6

**Published:** 2024-07-24

**Authors:** Jose Manuel Rivera Espejo, Sven De Maeyer, Steven Gillis

**Affiliations:** 1 Faculty of Social Sciences, Department of Training and Education Sciences, Antwerp University, Antwerp, Belgium; 2 Computational Linguistics and Psycholinguistics Research Centre CLIPS, University of Antwerp, Antwerp, Belgium

**Keywords:** Bayesian analysis, Speech intelligibility, Bounded outcomes, Clustering, Measurement error, Outliers, Heteroscedasticity, Generalized linear latent and mixed models, Robust regression models

## Abstract

When investigating unobservable, complex traits, data collection and aggregation processes can introduce distinctive features to the data such as boundedness, measurement error, clustering, outliers, and heteroscedasticity. Failure to collectively address these features can result in statistical challenges that prevent the investigation of hypotheses regarding these traits. This study aimed to demonstrate the efficacy of the Bayesian beta-proportion generalized linear latent and mixed model (beta-proportion GLLAMM) (Rabe-Hesketh et al.,* Psychometrika, *69(2), 167–90, 2004a, *Journal of Econometrics*, 128(2), 301–23, 2004c, 2004b; Skrondal and Rabe-Hesketh 2004) in handling data features when exploring research hypotheses concerning speech intelligibility. To achieve this objective, the study reexamined data from transcriptions of spontaneous speech samples initially collected by Boonen et al. (*Journal of Child Language*, 50(1), 78–103, 2023). The data were aggregated into entropy scores. The research compared the prediction accuracy of the beta-proportion GLLAMM with the normal linear mixed model (LMM) (Holmes et al., 2019) and investigated its capacity to estimate a latent intelligibility from entropy scores. The study also illustrated how hypotheses concerning the impact of speaker-related factors on intelligibility can be explored with the proposed model. The beta-proportion GLLAMM was not free of challenges; its implementation required formulating assumptions about the data-generating process and knowledge of probabilistic programming languages, both central to Bayesian methods. Nevertheless, results indicated the superiority of the model in predicting empirical phenomena over the normal LMM, and its ability to quantify a latent potential intelligibility. Additionally, the proposed model facilitated the exploration of hypotheses concerning speaker-related factors and intelligibility. Ultimately, this research has implications for researchers and data analysts interested in quantitatively measuring intricate, unobservable constructs while accurately predicting the empirical phenomena.

## Introduction

Intelligibility is at the core of successful, felicitous communication. Thus, being able to speak intelligibly is a major achievement in language acquisition and development. Moreover, intelligibility is considered to be the most practical index to assess competence in oral communication (Kent et al., 1994). Consequently, it serves as a key indicator for evaluating the effectiveness of various interventions like speech therapy or cochlear implantation (Chin et al., 2012).

The notion of speech intelligibility may appear deceptively simple, yet it is an intricate concept filled with inherent challenges in its assessment. Intelligibility refers to the extent to which a listener can accurately recover the words in a speaker’s acoustic signal (Freeman et al., 2017; van Heuven, 2008; Whitehill & Chau, 2004). Furthermore, achieving intelligible spoken language requires mastering all core components of speech perception, cognitive processing, linguistic knowledge, and articulation (Freeman et al., 2017). Hence, it is unsurprising that its accurate measurement faces challenges (Kent et al., 1989). These challenges arise from the interplay of the attributes of the communicative environment such as background noise (Munro, 1998), with features of the speaker like speaking rate (Munro & Derwing, 1998) or accent (Jenkins, 2000; Ockey et al., 2016), and characteristics of the listener like vocabulary proficiency or hearing ability (Varonis & Susan, 1985).

While several approaches have been proposed to assess intelligibility, they commonly rely on two types of speech samples: read-aloud or imitated, and spontaneous speech samples. Most studies favor read-aloud or imitated speech samples due to the substantial control they offer in selecting stimuli for intelligibility assessment. Additionally, these types of speech facilitate a direct and unambiguous comparison between a defined word target, produced by a speaker, and the listener’s identification of it, as exemplified by multiple studies such as Castellanos et al. (2014), Chin et al. (2012), Chin and Kuhns (2014), Freeman et al. (2017), Khwaileh and Flipsen (2010), and Montag et al. (2014). However, it has been demonstrated that these controlled speech samples exhibit limited efficacy in predicting intelligibility among hearing-impaired individuals (Cox et al., 1989; Ertmer, 2011). In contrast, spontaneous speech samples offer a more ecologically valid approach to assess intelligibility, resembling everyday informal speech more than read-aloud or imitated speech samples (Boonen et al., 2023). However, due to the uncertainty surrounding the speaker’s intended word production, it is unfeasible to establish a word target for these samples (Flipsen, 2006; Lagerberg et al., 2014). This renders conventional accuracy metrics from imitated speech, such as the percentage of read or imitated words, impractical (Boonen et al., 2023).

Yet, various metrics of intelligibility can still be derived from transcriptions of spontaneous speech samples, including the percentage of (un)intelligible words or syllables (Flipsen, 2006; Lagerberg et al., 2014), as well as entropy scores (Boonen et al., 2023). In the latter approach, listeners transcribe orthographically spontaneous speech samples produced by various speakers. These transcriptions are then aggregated into entropy scores, where lower scores indicate a higher degree of agreement among the listeners transcriptions and, consequently, higher intelligibility, while higher scores suggest lower intelligibility due to a lower degree of agreement in the transcriptions (Boonen et al., 2023; Faes et al., 2022). Notably, the aggregation procedure assumes that speech samples are considered intelligible if all listeners decode them in the same manner. These scores have been instrumental in examining differences in speakers’ speech intelligibility, particularly between children with normal hearing and those with cochlear implants (Boonen et al., 2023).

However, despite the entropy scores’ potential as a fine-grained metric of intelligibility, as proposed by Boonen et al. (2023), they exhibit a statistical complexity that cautions researchers against treating them as straightforward indices of intelligibility. This complexity emerges from the processes of data collection and transcription aggregation, endowing the scores with four distinctive features: boundedness, measurement error, clustering, and the possible presence of outliers and heteroscedasticity. Firstly, entropy scores are confined to the interval between 0 and 1, a phenomenon known as boundedness. Boundedness refers to the restriction of data values within specific bounds or intervals, beyond which they cannot occur (Lebl, 2022). Secondly, entropy scores are assumed to be a manifestation of a speaker’s intelligibility, with this intelligibility being the primary factor influencing the observed scores. This issue is commonly referred to as measurement error, signifying the disparity between the observed values of a variable, recorded under similar conditions, and some fixed *true value*, which is not directly observable (Everitt & Skrondal, 2010). Thirdly, due to the repeated assessment of speakers through multiple speech samples, the scores exhibit clustering. Clustering occurs when outcomes stem from repeated measurements of the same individual, location, or time (McElreath, 2020). Lastly, driven by speech samples with entropy scores located at the extreme of the bounds, and the presence of more than one population in the data (i.e., normal hearing versus hearing-impaired speakers), the scores may exhibit a potential for outliers and heteroscedasticity. Outliers are observations that markedly deviate from other sample data points where they occur (Grubbs, 1969), while heteroscedasticity occurs when the outcome’s variance depends on the values of another variable (Everitt & Skrondal, 2010).

Failure to collectively address these data features can result in numerous statistical challenges that might hamper the researcher’s ability to investigate intelligibility. Notably, neglecting boundedness can, at best, lead to underfitting and, at worst, to misspecification. Underfitting occurs when statistical models fail to capture the underlying data patterns, potentially generating predictions outside the data range, thus hindering the model’s ability to generalize when confronted with new data. Conversely, misspecification, which is marked by a poor representation of relevant aspects of the true data in the model’s functional form, can lead to inconsistent and less precise parameter estimates (Everitt & Skrondal, 2010). Additionally, overlooking issues such as measurement error, clustering, outliers, or heteroscedasticity can lead to biased and less precise parameter estimates (McElreath, 2020), ultimately diminishing the statistical power of models and increasing the likelihood of committing type I or type II errors when addressing research inquiries. Type I error results when the null hypothesis is falsely rejected, while type II error that results when the null hypothesis is falsely accepted (Everitt & Skrondal, 2010).

In computational statistics and data analysis, several models have been developed to address some of these data features individually and, at times, collectively. For instance, Ferrari and Cribari-Neto (2004) and Simas et al. (2010) initially introduced and expanded beta regression models to handle outcomes constrained within the unit interval. Subsequently, Figueroa-Zúñiga et al. (2013) extended these models to address data clustering. Over time, beta regression models have evolved to accommodate clustering and measurement errors in covariates, as demonstrated by Carrasco et al. (2012) and Figueroa-Zúñiga et al. (2018). Furthermore, robust versions of these models have been proposed to account for other statistical data issues, such as outliers and heteroscedasticity, as seen in Bayes et al. (2012) and Figueroa-Zúñiga et al. (2021). Robust models are a general class of statistical procedures designed to reduce the sensitivity of the parameter estimates to mild or moderate departures of the data from the model’s assumptions (Everitt & Skrondal, 2010). Ultimately, the work of Rabe-Hesketh and colleagues introduced the generalized linear latent and mixed model (GLLAMM) (Rabe-Hesketh et al., 2004a, 2004c, 2004b; Skrondal & Rabe-Hesketh, 2004), a unified framework that can simultaneously tackle all of the aforementioned data features.

All of these models have found moderate adoption in various fields, including speech communication (Boonen et al., 2023), psychology (Unlu & Aktas, 2017), cognition (Lopes et al., 2023; Verkuilen & Smithson, 2013), education (Pereira et al., 2020), health care (Ghosh, 2019; Kangmennaang et al., 2023), chemistry (de Brito Trindade et al., 2021), and policy analysis (Dieteren et al., 2023; Choi, 2023; Zhang et al., 2023). Specifically, in the domain of speech communication, Boonen et al. (2023) addressed data clustering within the context of intelligibility research. Conversely, de Brito Trindade et al. (2021) and Kangmennaang et al. (2023) concentrated on tackling non-normal bounded data with measurement error in covariates, within the context of chemical reactions and health care access, respectively. Remarkably, despite these individual efforts, there is, to the best of the authors’ knowledge, no study comprehensively addressing all of these data features in a principled way, while also transparently and systematically documenting the Bayesian estimation of the resulting statistical models.

This study employed Bayesian procedures for three main reasons. Firstly, prior research has consistently demonstrated the superiority of Bayesian methods over frequentist methods, especially with complex and overparameterized models (Baker, 1998; Kim & Cohen, 1999), such as the GLLAMM used in this study. Overparameterized models are those with more parameters than observations for estimation (Everitt & Skrondal, 2010). Secondly, the Bayesian approach enabled the incorporation of prior information, thereby constraining certain parameters within specified bounds. This feature addressed issues such as non-convergence or improper parameter estimation common in complex models under frequentist methods (Martin & McDonald, 1975; Seaman et al., 2011). An example is the estimation of negative variances for random effects in hierarchical models (Holmes et al., 2019), a problem resolved in this study through the utilization of prior distributions. Lastly, Bayesian methods have exhibited proficiency in drawing inferences from small sample sizes (Baldwin & Fellingham, 2013; Depaoli, 2014; Lambert et al., 2006). This feature of the Bayesian methods holds relevance for this study, as it also grapples with a small sample size, where reliance on the asymptotic properties of frequentist methods may not be justified.

## Research questions

Considering the imperative need to comprehensively address all features of the data when investigating unobservable and complex traits, this investigation aimed to demonstrate the efficacy of the generalized linear latent and mixed model (GLLAMM) in handling entropy score features when exploring research hypotheses concerning speech intelligibility. To achieve this objective, the study reexamined data originating from transcriptions of spontaneous speech samples, initially collected by Boonen et al. (2023). The data were aggregated into entropy scores and subjected to modeling through the Bayesian beta-proportion GLLAMM.

To address the primary objective, the study posed three key research questions. First, given the importance of accurate predictions in developing useful practical models and testing research hypotheses (Shmueli & Koppius, 2011), *Research Question 1 (RQ1)* evaluated whether the beta-proportion GLLAMM yielded more accurate predictions than the widely used normal linear mixed model (LMM) (Holmes et al., 2019). Second, acknowledging that intelligibility is an unobservable, intricate concept and a key indicator of oral communication competence (Kent et al., 1994), *Research Question 2 (RQ2)* investigated how the proposed model can estimate speakers’ latent intelligibility from manifest entropy scores. Thirdly, recognizing that research involves developing and comparing hypotheses, *Research Question 3 (RQ3)* illustrated how these research hypotheses can be examined within the model’s framework. Specifically, RQ3 assessed the influence of speaker-related factors on the newly estimated latent intelligibility.

Ultimately, this study offers researchers studying speech intelligibility through entropy scores and those in similar or different fields facing analogous data challenges with a statistical tool that improves upon current research models. This tool assess the predictability of empirical phenomena and develops a quantitative measure for the latent variable of interest. This quantitative measure, in turn, facilitates the appropriate comparison of existing hypotheses related to the latent variable, and even encourages the formulation of new ones.

## Methods

### Data

The data comprised the transcriptions of spontaneous speech samples originally collected by Boonen et al. (2023). The data is not publicly available due to privacy restrictions. Nonetheless, the data can be provided by the corresponding author upon reasonable request.

### Speakers

Boonen et al. (2023) selected $32$ speakers, comprising $16$ normal hearing children (NH) and $16$ hearing-impaired children with cochlear implants (HI/CI). At the time of the collection of the speech samples, the NH group was between $68$ and $104$ months old ($M=86.3$, $SD=9.0$), while HI/CI group were between $78$ and $98$ months old ($M=86.3$, $SD=6.7$). All children were native speakers of Belgian Dutch.

### Speech samples

Boonen and colleagues selected speech samples from a large corpus of children’s spontaneously spoken speech recordings. These recordings were made in Belgian Dutch and obtained while the children narrated a story prompted by the picture book “Frog, Where Are You?” (Mayer, 1969) to a caregiver ‘unfamiliar with the story’. Before the actual recording, the children were allowed to skim over the booklet and examine the pictures. Prior to the selection of the samples, the recordings were orthographically transcribed using the CHAT format in the CLAN editor (MacWhinney, 2020). These transcriptions were exclusively used in the selection of appropriate speech samples. To ensure the quality of the selection, Boonen and colleagues excluded sentences containing syntactically ill-formed or incomplete statements, with background noise, crosstalk, long hesitations, revisions, or non-words. Finally, ten speech samples were randomly chosen for each of the $32$ selected speakers. Each of these samples comprised a single sentence with a length of 3–11 words ($M=7.1$, $SD=1.1$). The process resulted in a total of $320$ selected sentences collectively comprising $2263$ words.

### Listeners

Boonen and colleagues recruited $105$ students from the University of Antwerp. All participants were native speakers of Belgian Dutch and reported no history of hearing difficulties or prior exposure to the speech of hearing-impaired speakers.

### Transcription task and entropy scores

Boonen et al. (2023) distributed the $320$ speech samples and $105$ listeners into five blocks through random allocation. Each block comprised $21$ listeners and $64$ sentences with no overlap between the blocks. The listeners were tasked with transcribing each sentence, which were presented to them in a random order. This resulted in a total of $\text{47,514}$ transcribed words from the original $2263$ words available in the speech samples. These orthographic transcriptions were automatically aligned with a Python script (Boonen et al., 2023), at the sentence level in a column-like grid structure like the one presented in Table 1. This alignment process was repeated for each sentence from every speaker, and the output was manually checked and adjusted (if needed) in order to appropriately align the words. For more details on the random assignment and alignment procedures refer to the original authors.

**Table 1:** Hypothetical alignment of word transcriptions and entropy scores. **Note:** Extracted from Boonen et al. (2023), and slightly modified for illustrative purposes. Entropy scores were calculated from words of the first sentence, produced by the first speaker assigned to the first block, and transcribed by five listeners $\left(s=1,i=1,b=1,J=5\right)$. Transcriptions are in Belgian Dutch followed by their English translation. [B] represent a blank space, and [X] an unidentifiable speech

Transcription	Words				
Number	1	2	3	4	5
1	de	jongen	ziet	een	kikker
	the	boy	sees	a	frog
2	de	jongen	ziet	de	[X]
	the	boy	sees	the	[X]
3	de	jongen	zag	[B]	kokkin
	the	boy	saw	[B]	cook
4	de	jongen	zag	geen	kikkers
	the	boy	saw	no	frogs
5	de	hond	zoekt	een	[X]
	the	dog	searches	a	[X]
Entropy	$0$	$0.3109$	$0.6555$	$0.8277$	$1$

Next, the aligned transcriptions were aggregated by listener, yielding $\text{2,263}$ entropy scores, one score per word for every sentence. The entropy scores were calculated following Shannon’s formula (1948):

1 $${H}_{wsib}=\frac{-\left[\left.{\sum }_{k=1}^{K}{p}_{k}\cdot {log}_{2}\left({p}_{k}\right)\right)\right.}{{log}_{2}\left(J\right)}$$

where ${H}_{\text{wsib}}$ denotes the entropy scores confined to an interval between zero and one, with $w$ defining the word index, $s$ the sentence index, $i$ the speaker index, and $b$ the block index. In addition, $K$ describes the number of different word types within transcriptions, and $J$ defines the total number of word transcriptions. Notice that by design, the total number of word transcriptions $J$ corresponds with the number of listeners per block, i.e., $21$ listeners. Lastly, ${p}_{k}={\sum }_{j=1}^{J}1\left({T}_{jk}\right)/J$ denotes the proportion of word types within transcriptions, with $1\left({T}_{jk}\right)$ describing an indicator function that takes the value of one when the word type $k$ is present in the transcription $j$. See Sect. Entropy scores calculation for an example of how entropy scores are computed.

These entropy scores served as the outcome variable, capturing agreement or disagreement among listeners’ word transcriptions. Lower scores indicated a higher degree of agreement between transcriptions and therefore higher intelligibility, while higher scores indicated lower intelligibility, due to a lower degree of agreement in the transcriptions (Boonen et al., 2023; Faes et al., 2022). Furthermore, no score was excluded from the modelling process using univariate procedures, rather, the identification of highly influential observations was performed within the context of the proposed models, as recommended by McElreath (2020).

### Statistical models

This section articulates the probabilistic formalism of both the normal LMM and the proposed beta-proportion GLLAMM. Subsequently, it details the set of fitted models and the estimation procedure, along with the criteria employed to assess the quality of the Bayesian inference results. Lastly, the section outlines the methodology employed for model comparison.

### Normal LMM

The general mathematical formalism of the normal LMM posits that the likelihood of the (manifest) entropy scores follow a normal distribution, i.e.

2 $${H}_{\text{wsib}}\sim {\text{Normal}}\left({\mu }_{\text{sib}},{\sigma }_{i}\right)$$

where ${\mu }_{\text{sib}}$ represents the average entropy at the word-level and ${\sigma }_{i}$ denotes the standard deviation of the average entropy at the word level, varying for each speaker. Given the clustered nature of the data, ${\mu }_{\text{sib}}$ is defined by the linear combination of individual characteristics and several random effects:

3 $${\mu }_{\text{sib}}=\alpha +{\alpha }_{HS\left[i\right)}+{\beta }_{A,HS\left[i\right)}\left({A}_{i}-\bar{A}\right)+{u}_{si}+{e}_{i}+{a}_{b}$$

where $H{S}_{i}$ and ${A}_{i}$ denote the hearing status and chronological age of speaker $i$, respectively. Additionally, $\alpha$ denotes the general intercept, ${\alpha }_{HS\left[i\right)}$ represents the average entropy for each hearing status group, and ${\beta }_{A,HS\left[i\right)}$ denotes the evolution of the average entropy per unit of chronological age ${A}_{i}$ for each hearing status group. Furthermore, ${u}_{si}$ denotes the sentence-speaker random effects measuring the unexplained entropy variability within sentences for each speaker, ${e}_{i}$ denotes the speaker random effects describing the unexplained entropy variability between speakers, and ${a}_{b}$ denotes the block random effects assessing the unexplained variability between experimental blocks.

Several notable features of the normal LLM can be discerned from the equations. Firstly, Eq. (2) indicates that the variability of the average entropy at the word level can differ for each speaker, enhancing the model *robustness* to mild or moderate data departures from the normal distribution assumption, such as in the presence of heteroscedasticity or outliers. Secondly, Eq. (3) reveals that the model assumes that no transformation is applied to the relationship between the average entropy and the linear combination of speakers’ characteristics. This is commonly known as a direct link function. In addition, the equation indicates that chronological age is *centered* around the minimum chronological age in the sample $\bar{A}$ . The *centering* procedure prevents the interpretation of parameters outside the range of chronological ages available in the data (Everitt & Skrondal, 2010). Also, the equation implies the model considers separate intercept and separate age slopes for each hearing status group, i.e., ${\alpha }_{HS\left[i\right)}$ and ${\beta }_{A,HS\left[i\right)}$ for NH and HI/CI speakers, respectively. Lastly, the presence of a general intercept $\alpha$ in the equation reveals that the model is overparameterized. Although the estimation of overparameterized models is only possible under Bayesian methods, their estimation does not violate any statistical principle (McElreath, 2020, 345). In contrast, in this study, the overparameterized model facilitates: (1) the comparison between the specific parameter interpretations of the normal LMM and the beta-proportion GLLAMM, with $\alpha$ serving no particular purpose in the former case, and (2) the assignment of prior distributions.

### *Beta*-proportion GLLAMM

The general mathematical formalism of the proposed beta-proportion GLLAMM comprises four components: a response model likelihood, a linear predictor, a link function, and a structural model. The likelihood of the response model posits that entropy scores follow a beta-proportion distribution,

4 $${H}_{\text{wsib}}\sim {\text{BetaProp}}\left({\mu }_{ib},{M}_{i}\right)$$

where ${\mu }_{ib}$ denotes the average entropy at the word-level and ${M}_{i}$ signifies the *dispersion* of the average entropy at the word-level, varying for each speaker. Additionally, ${\mu }_{ib}$ is defined as,

5 $${\mu }_{\text{ib}}={\text{logit}}^{-1}\left({a}_{b}-S{I}_{i}\right)$$

where ${\text{logit}}^{-1}\left(x\right)=exp\left(x\right)/\left(1+exp\left(x\right)\right)$ is the inverse-logit link function, ${a}_{b}$ denotes the block random effects, and $S{I}_{i}$ describes the speaker’s latent *potential intelligibility*. Conversely, the structural equation model relates the speakers’ latent potential intelligibility to the individual characteristics:

6 $$S{I}_{i}=\alpha +{\alpha }_{HS\left[i\right)}+{\beta }_{A,HS\left[i\right)}\left({A}_{i}-\bar{A}\right)+{e}_{i}+{u}_{i}$$

where $\alpha$ defines the general intercept, ${\alpha }_{HS\left[i\right)}$ denotes the potential intelligibility for different hearing status groups, and ${\beta }_{A,HS\left[i\right)}$ indicates the evolution of potential intelligibility per unit of chronological age for each hearing status group. Furthermore, ${e}_{i}$ represents speakers block effects, describing unexplained potential intelligibility variability between speakers, and ${u}_{i}={\sum }_{s=1}^{S}{u}_{si}/S$ denotes sentence random effects, assessing the average unexplained potential intelligibility variability within sentences for each speaker, with $S$ denoting the total number of sentences per speaker.

Several features are evident in the probabilistic representation of the model. Firstly, akin to the normal LMM, Eq. (4) reveals that the *dispersion* of average entropy at the word level can differ for each speaker. This enhances the model’s robustness to mild or moderate data departures from the beta-proportion distribution assumption. Secondly, in contrast to the normal LMM, Eq. (5) shows the potential intelligibility of a speaker has a negative non-linear relationship with the entropy scores. The negative relationship explicitly highlights the inverse relationship between intelligibility and entropy, while the non-linear relationship maps the unbounded linear predictor to the bounded limits of the entropy scores. Thirdly, in contrast with the normal LMM, Eq. (6) demonstrates that the structural parameters are interpretable in terms of the latent potential intelligibility scores, where the scale of the latent trait is set by the general intercept $\alpha$, as it is required in latent variable models (Depaoli, 2021). Furthermore, the equation implies the model also considers separate intercept and separate age slopes for each hearing status group, i.e., ${\alpha }_{HS\left[i\right)}$ and ${\beta }_{A,HS\left[i\right)}$ for NH and HI/CI speakers, respectively. In addition, it indicates that chronological age is *centered* around the minimum chronological age in the sample $\bar{A}$ . Lastly, the equation also reveals that the intelligibility scores have two sources of unexplained variability. The term ${e}_{i}$ represents inherent differences in potential intelligibility among different speakers. The term ${u}_{i}$ assumes that different sentences measure potential intelligibility differently due to variations in word difficulties and their interplay within the sentence.

### Prior distributions

Bayesian procedures require the incorporation of priors. Priors are probability distributions summarizing the information about known or assumed parameters prior to observing any empirical data (Everitt & Skrondal, 2010). Upon observing empirical data, these priors undergo updating to posterior distributions following Bayes’ rule (Jeffreys, 1998). In cases requiring greater modelling flexibility, a more refined representation of the parameters’ priors can be defined in terms of hyperparameters and hyperpriors. *Hyperparameters* refer to parameters indexing a family of possible prior distributions for the original parameter, while *hyperpriors* are prior distributions for such hyperparameters (Everitt & Skrondal, 2010).

This study established priors and hyperpriors for the parameters of both the normal LMM and the beta-proportion GLLAMM using prior predictive simulations. This procedure entails the semi-independent simulation of parameters, which are subsequently transformed into simulated data values according to the models’ specifications. The procedure aims to establish meaningful priors and comprehend their implications within the context of the model before incorporating any information derived from empirical data (McElreath, 2020). For reader inspection, the prior predictive simulations are provided in the accompanying digital walk-through document (see Sect. Open Science Statement).

### Normal LMM

For the parameters of the normal LMM, non-informative priors and hyperpriors were established to align with analogous model assumptions in frequentist methods. A *non-informative* prior reflects the distributional commitment of a parameter to a wide range of values within a specific parameter space (Everitt & Skrondal, 2010). The specified priors were as follows:

7 $$\begin{array}{cc}{r}_{S}& \sim {\text{Exponential}} \, \left(2\right)\\ {\sigma }_{i}& \sim {\text{Exponential}} \, \left({r}_{S}\right)\\ {m}_{i}& \sim {\text{Normal}} \, \left(\text{0,0.05}\right)\\ {s}_{i}& \sim {\text{Exponential}} \, \left(2\right)\\ {e}_{i}& \sim {\text{Normal}} \, \left({m}_{i},{s}_{i}\right)\\ {m}_{b}& \sim {\text{Normal}} \, \left(\text{0,0.05}\right)\\ {s}_{b}& \sim {\text{Exponential}} \, \left(2\right)\\ {a}_{b}& \sim {\text{Normal}} \, \left({m}_{b},{s}_{b}\right)\\ \alpha & \sim {\text{Normal}} \, \left(\text{0,0.05}\right)\\ {\alpha }_{HS\left[i\right)}& \sim {\text{Normal}} \, \left(\text{0,0.2}\right)\\ {\beta }_{A,HS\left[i\right)}& \sim {\text{Normal}} \, \left(\text{0,0.1}\right)\end{array}$$

### *Beta*-proportion GLLAMM

For the parameters of the beta-proportion GLLAMM, weakly informative priors and hyperpriors were established. *Weakly informative priors* reflect the distributional commitment of a parameter to a weakly constraint range of values within a realistic parameter space (McElreath, 2020). The specified priors were as follows:

8 $$\begin{array}{cc}{r}_{M}& \sim {\text{Exponential}} \, \left(2\right)\\ {M}_{i}& \sim {\text{Exponential}} \, \left({r}_{M}\right)\\ {m}_{i}& \sim {\text{Normal}} \, \left(\text{0,0.05}\right)\\ {s}_{i}& \sim {\text{Exponential}} \, \left(2\right)\\ {e}_{i}& \sim {\text{Normal}} \, \left({m}_{i},{s}_{i}\right)\\ {m}_{b}& \sim {\text{Normal}} \, \left(\text{0,0.05}\right)\\ {s}_{b}& \sim {\text{Exponential}} \, \left(2\right)\\ {a}_{b}& \sim {\text{Nor}}{\text{mal}} \, \left({m}_{b},{s}_{b}\right)\\ \alpha & \sim {\text{Normal}} \, \left(\text{0,0.05}\right)\\ {\alpha }_{HS\left[i\right)}& \sim {\text{Normal}} \, \left(\text{0,0.3}\right)\\ {\beta }_{A,HS\left[i\right)}& \sim {\text{Normal}} \, \left(\text{0,0.1}\right)\end{array}$$

### Fitted models

This study evaluated the comparative predictive capabilities of both the normal LMM and the beta-proportion GLLAMM (RQ1) while simultaneously examined various formulations regarding how speaker-related factors influence intelligibility (RQ3). In this context, the predictive capabilities of the models were intricately connected to these formulations. As a result, the study required fitting $12$ different models, each representing a specific manner to investigate one or both research questions. The models comprised six versions of both the normal LMM and the beta-proportion GLLAMM. The differences among the models hinged on (1) whether they addressed data clustering in conjunction with measurement error, denoted as the model type, (2) the assumed distribution for the entropy scores, which aimed to handle boundedness, (3) whether the model incorporated a robust feature to address mild or moderate departures of the data from distributional assumptions, and (4) the inclusion or exclusion of speaker-related factors in the models. A detailed overview of the fitted models is available in Table 2.

**Table 2:** Fitted models. **Note:** Yes indicates the feature or parameter is included in the model

	Model	Entropy	Robust	Fixed effects		
Model	type	distribution	feature	${\beta }_{HS\left[i\right]}$	${\beta }_{A}$	${\beta }_{A,HS\left[i\right]}$
1	LMM	Normal	No	No	No	No
2	LMM	Normal	No	Yes	Yes	No
3	LMM	Normal	No	Yes	No	Yes
4	LMM	Normal	Yes	No	No	No
5	LMM	Normal	Yes	Yes	Yes	No
6	LMM	Normal	Yes	Yes	No	Yes
7	GLLAMM	BetaProp	No	No	No	No
8	GLLAMM	BetaProp	No	Yes	Yes	No
9	GLLAMM	BetaProp	No	Yes	No	Yes
10	GLLAMM	BetaProp	Yes	No	No	No
11	GLLAMM	BetaProp	Yes	Yes	Yes	No
12	GLLAMM	BetaProp	Yes	Yes	No	Yes

### Estimation and chain quality

The models were estimated using R version 4.2.2 (R Core Team, 2015) and Stan version 2.26.1 (Stan Development Team. 2021). Four Markov chains were implemented for each parameter, each with distinct starting values. Each chain underwent $4000$ iterations, where the first $2000$ serving as a warm-up phase and the remaining $2000$ were considered samples from the posterior distribution. Verification of stationarity, convergence, and mixing for the parameter chains involved graphical analysis and diagnostic statistics. Graphical analysis utilized trace, trace-rank, and autocorrelation plots (ACF). Diagnostic statistics included the *potential scale reduction factor statistics* $\widehat{R}$ with a cut-off value of $1.05$ (Vehtari et al., 2021). Furthermore, to confirm whether the parameters posterior distributions were generated with a sufficient number of uncorrelated sampling points, each posterior distribution density plot was inspected along with their effective sample size statistics ${n}_{\text{eff}}$ (Gelman et al., 2014).

In general, both graphical analysis and diagnostic statistics indicated that all chains exhibited low to moderate autocorrelation, explored the parameter space in a seemingly random manner, and converged to a constant mean and variance in their post-warm-up phase. Moreover, the density plots and statistics collectively confirmed that all posterior distributions were unimodal distributions with values centered around a mean, generated with a satisfactory number of uncorrelated sampling points, making substantive sense compared to the models’ prior beliefs. The trace, trace-rank, ACF, and distribution density plots, along with $\widehat{R}$ and ${n}_{\text{eff}}$ statistics, are provided in the accompanying digital walk-through document for reader inspection (see Sect. Open Science Statement).

### Model comparison

This study compared the fitted models using three criteria: the deviance information criterion (DIC) introduced by Spiegelhalter et al. (2002), the widely applicable information criterion (WAIC) proposed by Watanabe (2013), and the Pareto Smoothing Importance Sampling criterion (PSIS) developed by Vehtari et al. (2017). These criteria score models in terms of deviations from *perfect* predictive accuracy, with smaller values indicating less deviation (McElreath, 2020). Deviations from *perfect* predictive accuracy serve as the closest estimate for the Kullback–Leibler divergence (Kullback & Leibler, 1951), which measures the degree to which a probabilistic model accurately represents the *true* distribution of the data. Specifically, DIC measures in-sample deviations, while WAIC and PSIS offer an approximate measure of out-of-sample deviations.

WAIC and PSIS are regarded as full Bayesian criteria because they encompass all the information contained in the parameter’s posterior distribution, effectively integrating and reporting the inherent uncertainty in predictive accuracy estimates. In addition to predictive accuracy, PSIS offers an extra benefit by identifying highly influential data points. To achieve this, the criterion employs a built-in warning system that flags observations that make out-of-sample predictions unreliable. The rationale is that observations that are relatively unlikely, according to the model, exert more influence and render predictions less reliable compared to those that are relatively expected (McElreath, 2020).

However, since researchers are mostly interested in comparing candidate models, it is the distance between the models that is useful, rather than the absolute value of the criteria (see McElreath, 2020, 209, 223–24). Therefore, this study utilized the differences in WAIC and PSIS (dWAIC and dPSIS, respectively) to evaluate how distinct our probabilistic models are from each other, and which one is closer to the *true* distribution of the data. Additionally, while DIC, WAIC and PSIS provide *approximately correct* estimates for the expected accuracy, the criteria are also subject to uncertainty due to the specific sample over which they are computed (see McElreath, 2020, 223). Thus, this uncertainty should also be taken into account for the criteria and their comparisons. Consequently, this study also presented the associated uncertainty for both criteria calculated as WAIC $\pm 1\cdot$ SE, PSIS $\pm 1\cdot$ SE, dWAIC $\pm 1\cdot$ dSE and dPSIS $\pm 1\cdot$ dSE. Lastly, this research also reported the models’ complexity penalization, as well as their associated weight of evidence. The complexity penalization values pWAIC and pPSIS are roughly associated with the models’ number of parameters, while the weight of evidence summarizes the relative support for each model.

### Open science statement

In an effort to improve the transparency and replicability of the analysis, this study provides access to an online walk-through. The digital document contains all the code and materials utilized in the study. Furthermore, the walk-through meticulously follows the When-to-Worry-and-How-to-Avoid-the-Misuse-of-Bayesian-Statistics checklist (WAMBS checklist) developed by Depaoli and van de Schoot (2017). This checklist outlines the ten crucial points that need careful scrutiny when employing Bayesian inference procedures. The digital walk-through is available at the following URL: https://jriveraespejo.github.io/paper1_manuscript/

## Results

This section presents the results of the Bayesian inference procedures, with particular emphasis on answering the three research questions.

### Predictive capabilities of the *beta*-proportion GLLAMM compared to the normal LMM (RQ1)

This research question evaluated the effectiveness of the beta-proportion GLLAMM in handling the features of entropy scores by comparing its predictive accuracy to the normal LMM. Models $1$, $4$, $7$, and $10$ were specifically chosen for this comparison because their assumptions exclusively addressed the features of the scores, without integrating additional covariate information. As detailed in Table 2, Model $1$ was a normal LMM that solely addresses data clustering. Building upon this, Model $4$ introduced a robust feature. Conversely, Model $7$ was a beta-proportion GLLAMM that deals with boundedness, measurement error and data clustering, and Model $10$ extended this model by incorporating a robust feature.

**Fig. 1:**
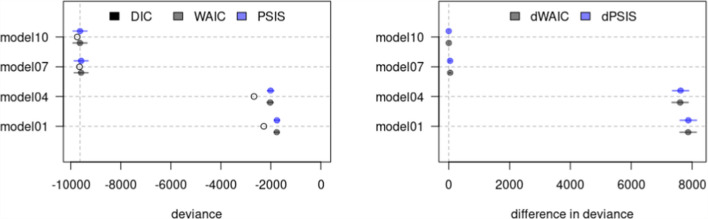
Comparison plot for selected models. **Note:** Open, black, and blue points describe the posterior means for the criteria. Continuous colored horizontal lines indicate the criteria associated uncertainty

The left panel of Fig. 1 displays the models’ DIC, WAIC, and PSIS values with their corresponding uncertainty intervals. In contrast, the right panel of the figure shows the models’ dWAIC and dPSIS values with their corresponding uncertainty intervals. Tables 6 and 7 provide similar information, while also reporting the pWAIC and pPSIS values and the weight of evidence for each model. Overall, all criteria consistently pointed to Model $10$ as the most plausible choice for the data. The model exhibits the lowest values for both WAIC and PSIS, establishing itself as the model with the least deviation from *perfect* predictive accuracy among those under comparison. Additionally, Fig. 1 visually demonstrates the non-overlapping uncertainty in both dWAIC and dPSIS values for Models $1$, $4$, and $7$ when compared to Model $10$. This indicates that Model $10$ significantly deviated the least from *perfect* predictive accuracy when compared to the rest of the models. Lastly, the weight of evidence in Tables 6 and 7 underscored that $100\%$ of the evidence aligned with and supported Model $10$.

**Fig. 2:**
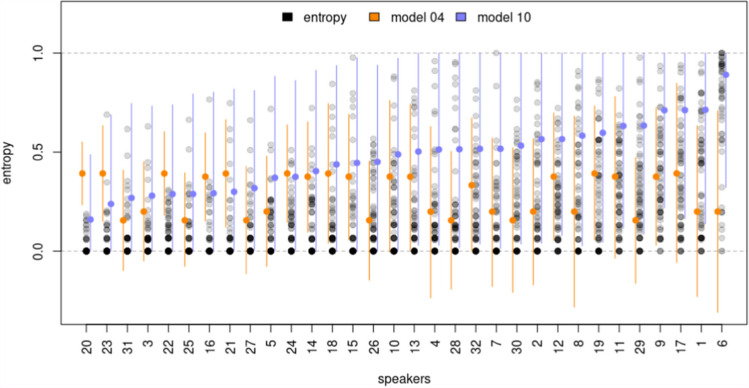
Entropy scores prediction for selected models. **Note:** Black points show manifest entropy scores where darker points indicate greater overlap. Orange dots and vertical lines show the posterior mean and 95% HPDI derived from Model 4. Blue dots and vertical lines show similar information from Model 10

Upon closer examination, the reasons behind the observed disparities in the models become more apparent. Specifically, Fig. 2 demonstrates that the normal LMM, as outlined in Model $4$, failed to adequately capture the data’s underlying patterns, resulting in predictions that were physically inconsistent. This issue is illustrated by the $95\%$ highest probability density intervals (HPDI) extending beyond the expected zero to one outcome range. Further insight into this lack of fit is provided by Fig. 9. The figure displays score prediction densities for Model $4$ that bore no resemblance to the actual data densities. Furthermore, the top two panels in Fig. 11 reveal that misspecification in the normal LMM caused the model to be *more surprised* by extreme entropy scores, leading to their identification as highly unlikely and influential observations. Consequently, the model was rendered unreliable due to the potential biases present in the parameter estimates. In contrast, the beta-proportion GLLAMM appeared to effectively capture the data patterns, generating predictions within the expected data range. This is evident in Fig. 2 and complemented by Figs. 10 and 11. In Fig. 10, Model $10$ displayed prediction densities that bore more resemblance to the actual data densities. Furthermore, the bottom two panels in Fig. 11 show the model was *less surprised* by extreme scores, fostering more trust in the model’s estimates.

### Estimation of speakers’ latent potential intelligibility from manifest entropy scores (RQ2)

The second research question aimed to demonstrate the application of the beta-proportion GLLAMM in estimating the latent potential intelligibility of speakers. This was achieved by employing the general mathematical formalism outlined in Eq. (6), along with additional specifications provided in Table 2. The Bayesian procedure successfully estimated the latent potential intelligibility of speakers under Model $10$ through the following structural equation:

9 $$S{I}_{i}=\alpha +{e}_{i}+{u}_{i}$$

Moreover, due to its implementation under Bayesian procedures, Model $10$ provided the complete posterior distribution of the speakers’ potential intelligibility scores. This provision, in turn, (1) enabled the calculation of summaries, facilitating the ranking of individuals, and (2) supported the assessment of differences among selected speakers. In both cases, the model considered the inherent uncertainty of the estimates resulting from its measurement using multiple entropy scores.

**Fig. 3:**
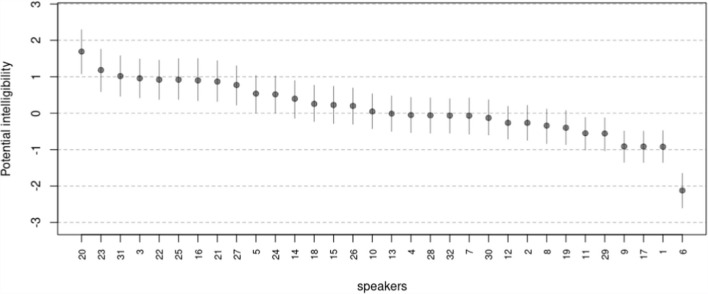
Model 10, latent potential intelligibility of speakers. **Note:** Black dots and vertical lines show the posterior means and 95% HPDI intervals

**Fig. 4:**
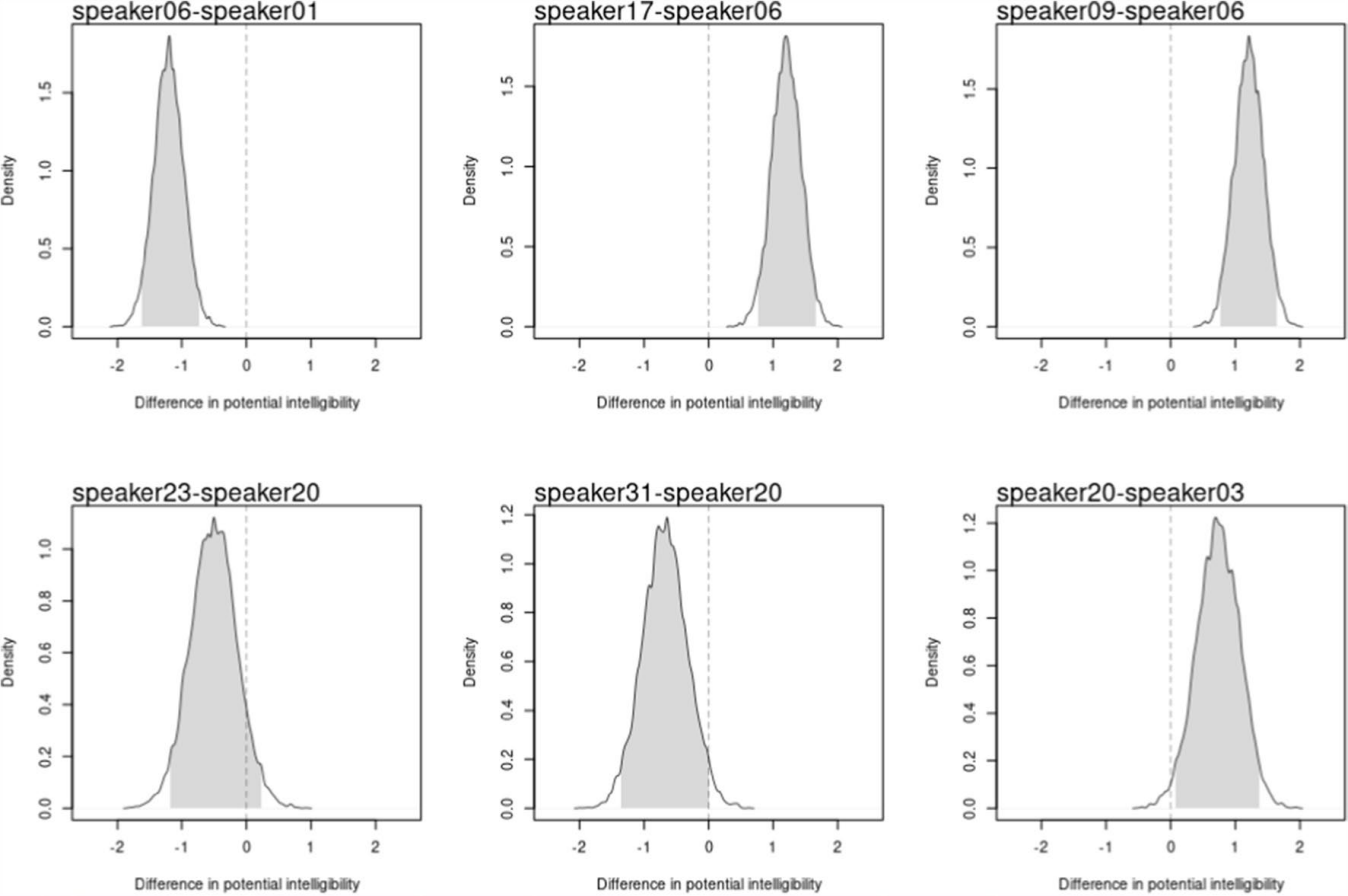
Model 10, potential intelligibility comparisons among selected speakers. **Note:** Shaded area describes the 95% HPDI

Figure 3 displays the ranking of speakers in decreasing order based on the posterior means of the latent potential intelligibility. These estimates are accompanied by their associated $95\%$ HPDI. The figure indicates that speaker $6$ stands out as the least intelligible in the sample, followed further behind by speaker $1$, $17$, and $9$. In contrast, the figure highlights speaker $20$ as the most intelligible, closely followed by speakers $23$, $31,$ and $3$. Conversely, the full posterior distribution for comparing potential intelligibility between the least and most intelligible speakers against other selected speakers is shown in Fig. 4. The figure reveals that only the differences between speakers $6$, $1$, $17$, and $9$, along with the difference between speakers $20$ and $3$ are statistically significant, as their associated $95\%$ HPDI did not overlap with zero (shaded area). The R code to derive these scores and generate the figure is available in the digital walk-through document (see Sect. 2.3 Open Science Statement).

### Testing the influence of speaker-related factors on intelligibility (RQ3)

This research question illustrated how hypotheses on intelligibility can be examined within the model’s framework. Specifically, the focus centered on assessing the influence of speaker-related factors on intelligibility, such as chronological age and hearing status. Notably, despite RQ1 indicating the suitability of the beta-proportion GLLAMM for entropy scores, existing statistical literature suggests that, in certain scenarios, models incorporating covariate adjustment exhibit robustness to misspecification in the functional form of the covariate-outcome relationship (Tackney et al., 2023). Consequently, this study compared all models detailed in Table 2. These models were characterized by different covariate adjustments on entropy scores or the latent potential intelligibility of speakers, namely chronological age and hearing status. Furthermore, some models like the normal LMMs, potentially exhibited misspecification in the covariate-outcome relationship.

**Fig. 5:**
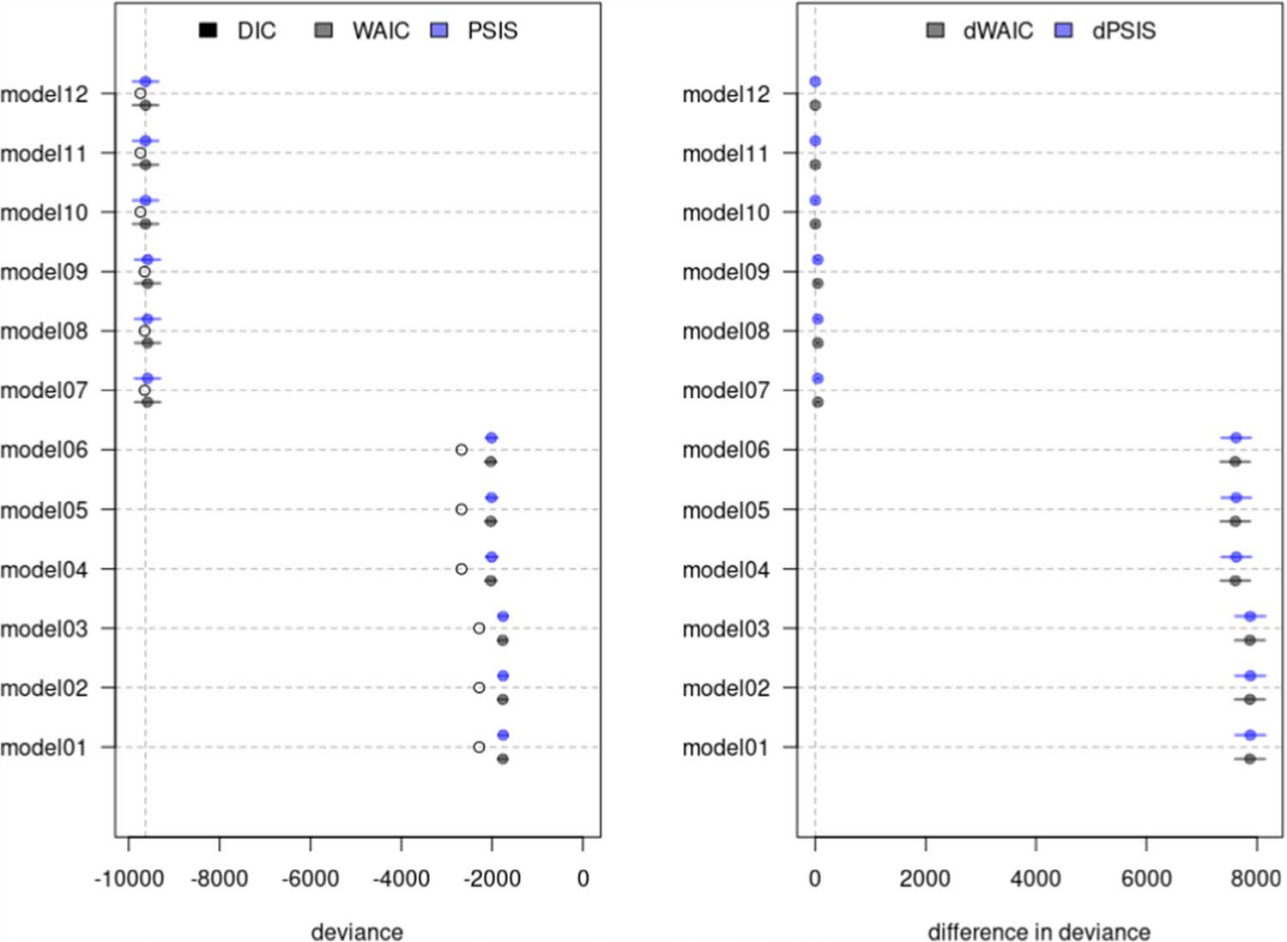
Comparison plot for all models. **Note:** Open, black, and blue points describe the posterior means for the criteria. Continuous colored horizontal lines indicate the criteria associated uncertainty

Similar to RQ1, all criteria consistently identified the beta-proportion GLLAMM outlined in models $12$, $11$, and $10$ as the most plausible models for the data. The models exhibited the lowest values for both WAIC and PSIS, establishing them as the least deviating models among those under comparison. In addition, Fig. 5 depicts the non-overlapping uncertainty for the models’ dWAIC and dPSIS values with horizontal blue lines. This reveals that, when compared to Model $12$, most models exhibited significantly distinct predictive capabilities. Models $11$ and $10$, however, stood out as exceptions to this pattern. This observation suggests that Models $12$, $11$, and $10$ displayed the least deviation from *perfect* predictive accuracy in contrast to the other models. Lastly, the weight of evidence in Tables 8 and 9, underscored that Model $11$ accumulated the greatest support, followed by Model $12$, and lastly, by Model $10$.

A closer examination of two models within this comparison set reveals the reasons behind the largest observed disparities. The normal LMM, as outlined in Model $6$, continued to face challenges in capturing underlying data patterns, resulting in predictions that are physically inconsistent, falling outside the outcome’s range. Additionally, the model persisted in identifying highly unlikely and influential observations, making it inherently unreliable. In contrast, the beta-proportion GLLAMM described by Model $12$ appeared to be less susceptible to extreme scores, effectively capturing data patterns within the expected data range and thereby instilling greater confidence in the reliability of the model’s estimates. This contrast is visually depicted in Figs. 12, 13, 14, 15.

Considering the results in Fig. 5, the model comparisons favored three distinct models: Models $10$, $11$, and $12$. Model $10$, supported by $20.4\%$ of the evidence, estimated a single intercept $\alpha$ and no slope to explain the potential intelligibility of speakers (see Table 3). In contrast, supported by $45.1\%$ of the evidence, Model $11$ in Table 4 estimated distinct intercepts for each hearing status group, namely ${\alpha }_{HS\left[1\right]}$ for NH speakers and ${\alpha }_{HS\left[2\right]}$ for the HI/CI counterparts, while maintaining a single slope that gauges the impact of age on potential intelligibility estimates. The $95\%$ HPDI for the comparison of intercepts ${\alpha }_{HS\left[2\right]}-{\alpha }_{HS\left[1\right]}$ revealed significant differences between NH and HI/CI speakers. Lastly, with evidence of $34.1\%$, Model $12$ in Table 5 estimated different intercepts and slopes per hearing status group, namely ${\alpha }_{HS\left[1\right]}$ and ${\beta }_{A,HS\left[1\right]}$ for the NH speakers, and ${\alpha }_{HS\left[2\right]}$ and ${\beta }_{A,HS\left[2\right]}$ for the HI/CI counterparts. The $95\%$ HPDI for the comparison of intercepts and slopes revealed significant differences solely in the slopes between NH and their HI/CI counterparts $\left({\beta }_{A,HS\left[2\right]}-{\beta }_{A,HS\left[1\right]}\right)$.

**Table 3:** Model 10, parameter estimates and 95% HPDI

Parameter	Posterior mean	95% HPDI
$\alpha$	0.01	[– 0.09, 0.1]

**Table 4:** Model 11, parameter estimates and 95% HPDI

Parameter	Posterior mean	95% HPDI
$\alpha$	0.01	[– 0.08, 0.11]
${\alpha }_{HS\left[1\right]}$	0.53	[0.11, 0.94]
${\alpha }_{HS\left[2\right]}$	– 0.03	[– 0.43, 0.39]
${\beta }_{A}$	0.07	[0.05, 0.10]
Contrasts		
${\alpha }_{HS\left[2\right]}-{\alpha }_{HS\left[1\right]}$	– 0.55	[– 1.00, – 0.15]

**Table 5:** Model 12, parameter estimates and 95% HPDI

Parameter	Posterior mean	95% HPDI
$\alpha$	0.01	[– 0.09, 0.11]
${\alpha }_{HS\left[1\right]}$	0.21	[– 0.28, 0.72]
${\alpha }_{HS\left[2\right]}$	0.23	[– 0.24, 0.69]
${\beta }_{A,HS\left[1\right]}$	0.10	[0.07, 0.13]
${\beta }_{A,HS\left[2\right]}$	0.06	[0.03, 0.09]
Contrasts		
${\alpha }_{HS\left[2\right]}-{\alpha }_{HS\left[1\right]}$	0.01	[– 0.61, 0.74]
${\beta }_{A,HS\left[2\right]}-{\beta }_{A,HS\left[1\right]}$	– 0.04	[– 0.08, 0.00]

**Fig. 6:**
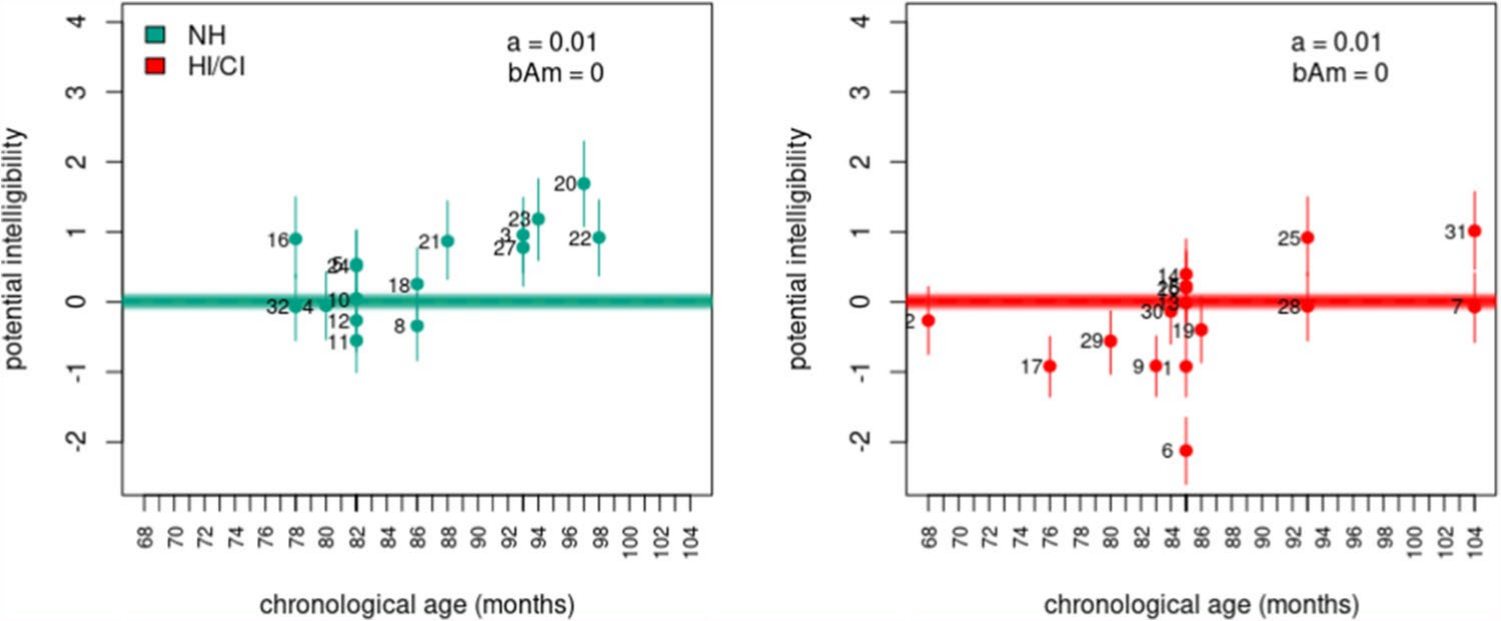
Model 10, potential intelligibility per chronological age and hearing status. **Note:** Colored dots denote the posterior means, vertical lines describe the 95% HPDI, thick discontinuous line indicates the regression line, thin continuous lines denote regression lines samples from the posterior distribution, and numbers indicate the speaker index

**Fig. 7:**
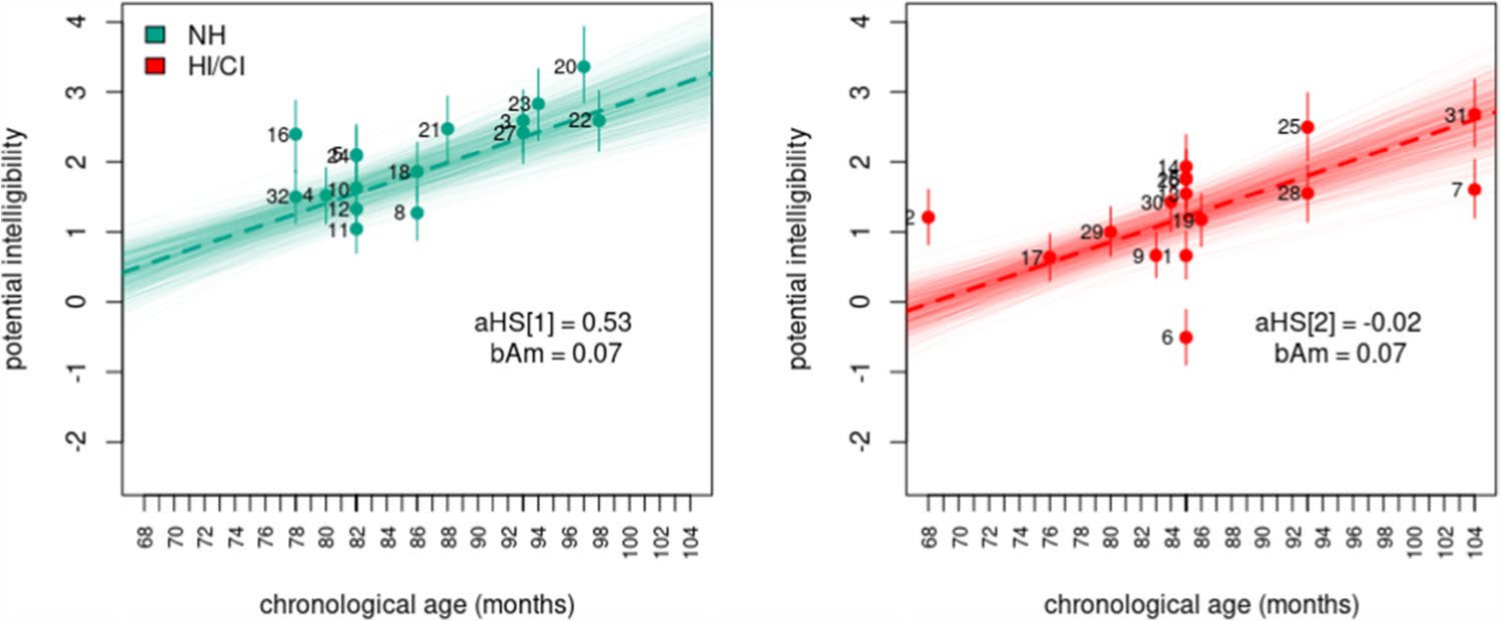
Model 11, Potential intelligibility per chronological age and hearing status. **Note:** Colored dots denote the posterior means, vertical lines describe the 95% HPDI, thick discontinuous line indicates the regression line, thin continuous lines denote regression lines samples from the posterior distribution, and numbers indicate the speaker index

**Fig. 8:**
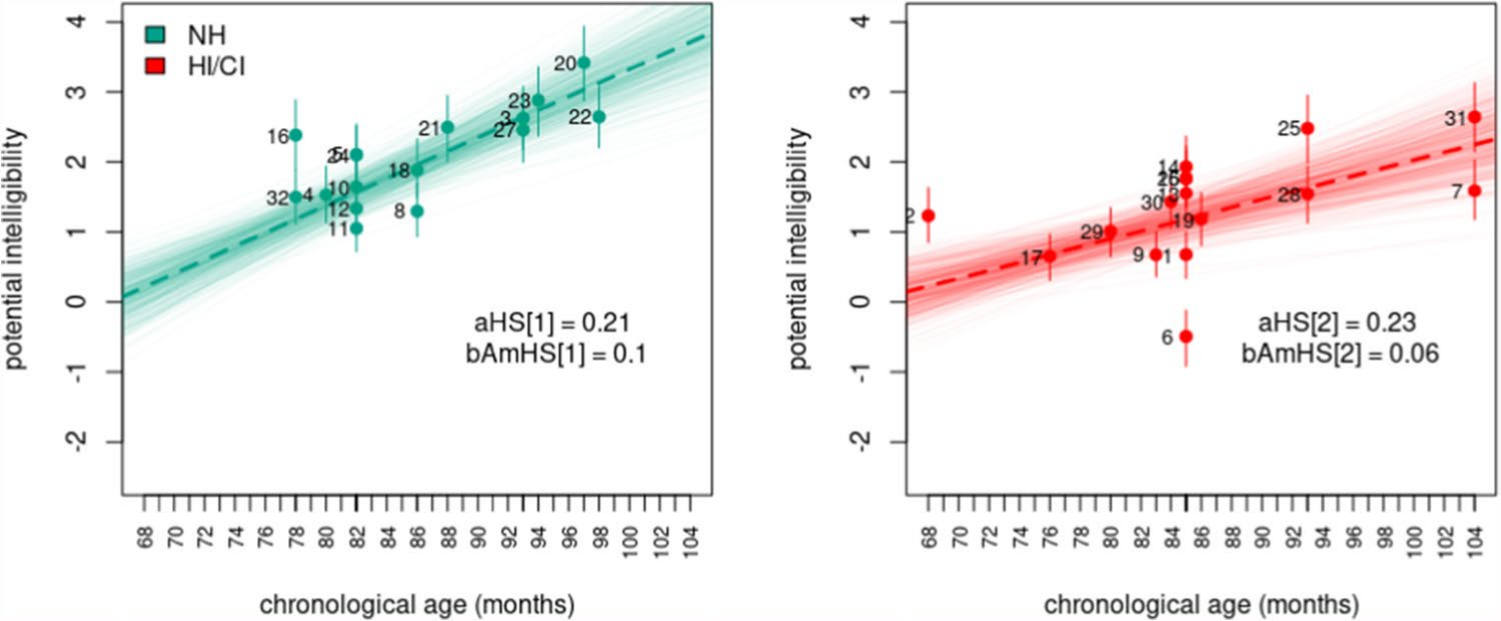
Model 12, potential intelligibility per chronological age and hearing status. **Note:** Colored dots denote the posterior means, vertical lines describe the 95% HPDI, thick discontinuous line indicates the regression line, thin continuous lines denote regression lines samples from the posterior distribution, and numbers indicate the speaker index

**Fig. 9:**
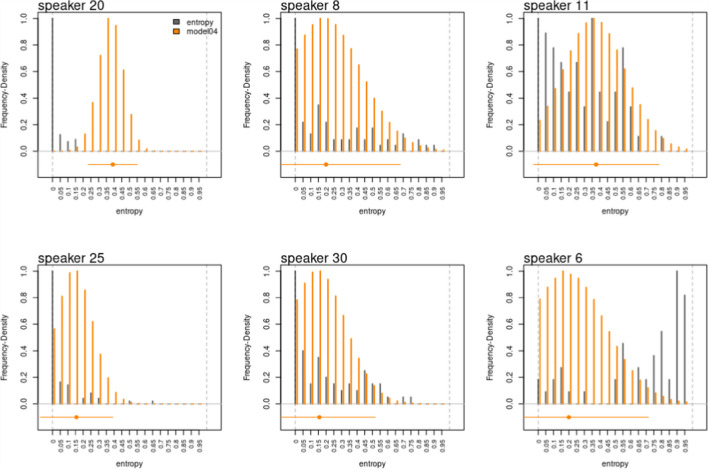
Model 4: Entropy scores density for selected speakers. **Note:** *Black bars* denote the true data density, *orange bars* describe the predicted data density

**Fig. 10:**
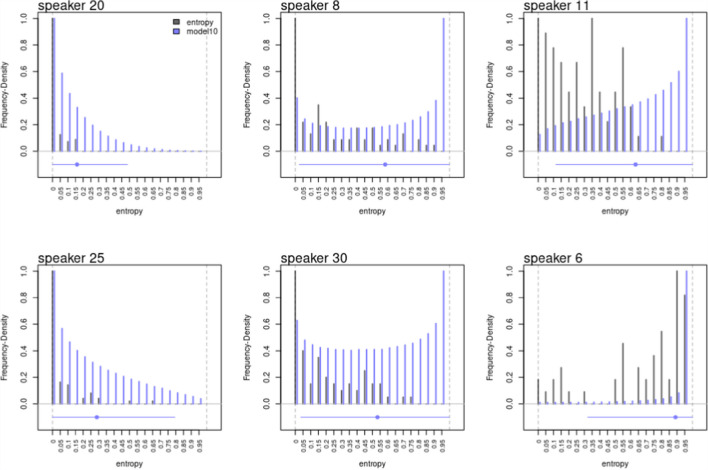
Model 10: Entropy scores density for selected speakers. **Note:** *Black bars* denote the true data density, *blue bars* describe the predicted data density

**Fig. 11:**
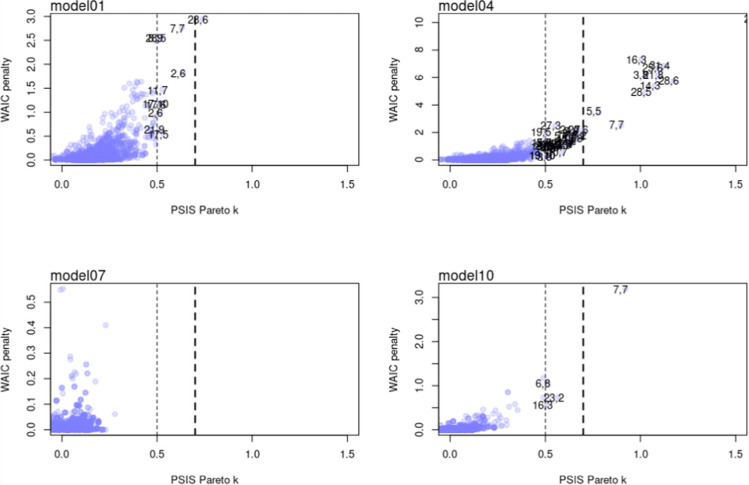
Outlier identification and analysis for selected models. **Note:** *Thin* and *thick vertical discontinuous lines* indicate threshold of 0.5 and 0.7, respectively. *Number pair texts* indicate the observation pair of speaker and sentence index

**Fig. 12:**
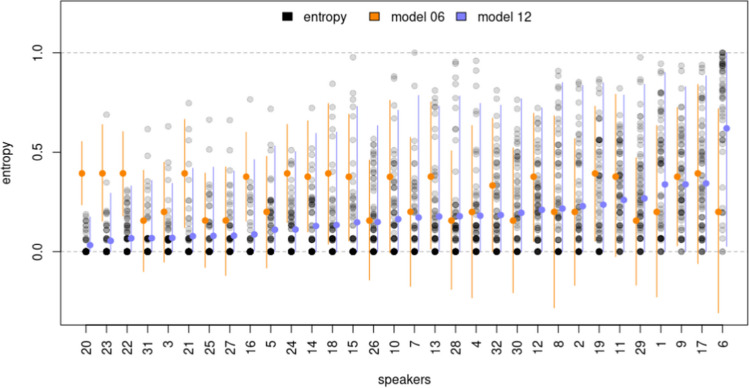
Entropy scores prediction for selected models. **Note:** *Black points* show manifest entropy scores where *darker points* indicate greater overlap. *Orange dots* and *vertical lines* show the posterior mean and 95% HPDI derived from Model 6. *Blue dots* and *vertical lines* show similar information from Model 12

**Fig. 13:**
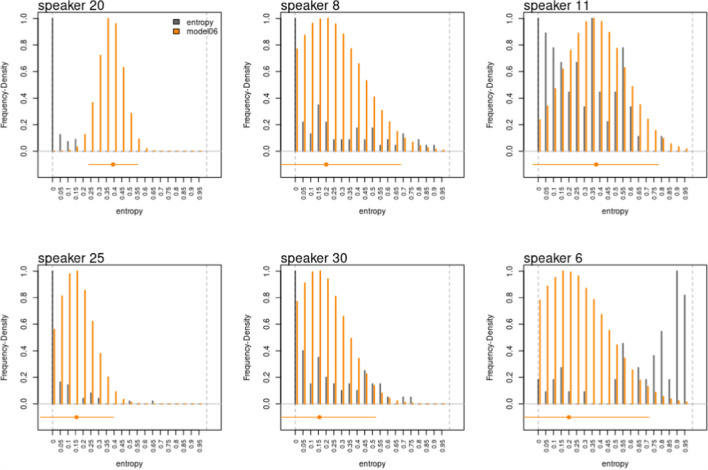
Model 6: Entropy scores density for selected speakers. **Note:** *Black bars* denote the true data density, *orange bars* describe the predicted data density

**Fig. 14:**
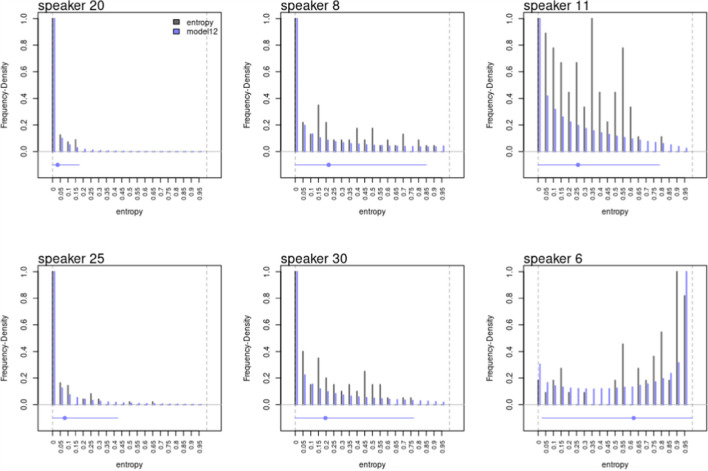
Model 12: Entropy scores density for selected speakers. **Note:** *Black bars* denote the true data density, *blue bars* describe the predicted data density

**Fig. 15:**
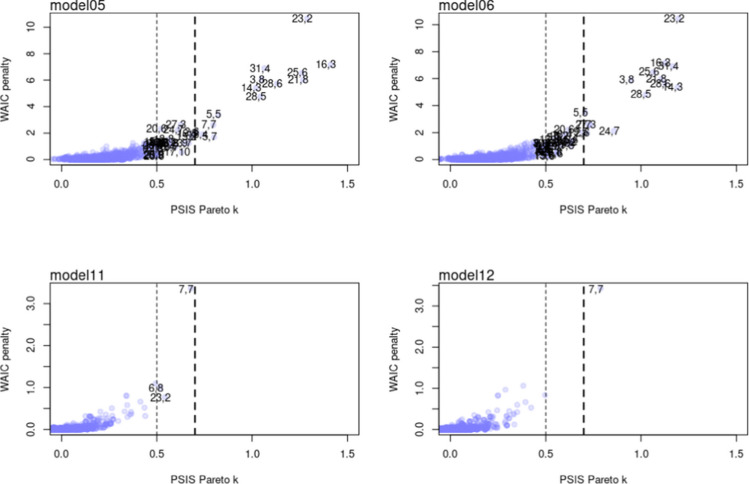
Outlier identification and analysis for selected models. **Note:** *Thin* and *thick vertical discontinuous lines* indicate threshold of 0.5 and 0.7, respectively. *Number pair texts* indicate the observation pair of speaker and sentence index

However, a discerning reader can notice that these models yielded conflicting conclusions regarding the influence of chronological age and hearing status on intelligibility. Model $10$ implied no influence of chronological age and hearing status on the potential intelligibility of speakers. Figure 6, however, revealed the reason for the model’s low support. Model $10$ failed to capture the prevalent increasing age pattern observed in potential intelligibility estimates. In contrast, Model $11$ identified significant differences in potential intelligibility between NH and HI/CI speakers. The model further suggested that with the progression of chronological age, HI/CI speakers lag behind in intelligibility development, with no opportunity to catch up to their NH counterparts within the analyzed age range, as depicted in Fig. 7. Finally, Model $12$ indicated no significant differences in intelligibility between NH and HI/CI speakers at $68$ months of age (around $6$ years old). However, the model revealed distinct evolution patterns of intelligibility per unit of chronological age between different hearing status groups, with HI/CI speakers displaying a slower rate of development compared to their NH counterparts within the analyzed age range. The latter is evident in Fig. 8.

## Discussion

### Findings

This study examined the suitability of the Bayesian beta-proportion GLLAMM for the quantitative measuring and testing of research hypotheses related to speech intelligibility using entropy scores. The initial findings supported the assertion that beta-proportion GLLAMMs consistently outperformed normal LMMs in predicting entropy scores, underscoring its superior predictive performance. The results also emphasized that models neglecting measurement error and boundedness in the outcomes lead to underfitting and misspecification issues, even when robust features are integrated. This was clearly illustrated by the normal LMMs.

Secondly, the study showcased the beta-proportion GLLAMM’s proficiency in estimating the latent potential intelligibility of speakers based on manifest entropy scores. Implemented under Bayesian procedures, the proposed model offered a valuable advantage over frequentist methods by further providing the full posterior distribution of the speakers’ potential intelligibility. This provision facilitated the calculation of summaries, aiding in the construction of individual rankings, and supported the comparisons among selected speakers. In both scenarios, the proposed model accounted for the inherent uncertainty in the intelligibility estimates.

Thirdly, the study illustrated how the proposed model assessed the impact of speaker-related factors on potential intelligibility. The results suggested that multiple models were plausible for the observed entropy scores. This indicated that different speaker-related factor hypotheses were viable for the data, with some presenting contradictory conclusions about the influence of these factors on intelligibility. However, even without unequivocal support for one hypothesis, the divided support among these models informed that certain statistical issues may be hindering the models’ ability to distinguish among individuals and, ultimately, among models. These issues may be attributed to factors such as the insufficient sample size of speakers, the inadequate representation of the population of speakers, referred to as selection bias, and the imprecise measurement of the latent variable of interest.

Ultimately, this study introduced researchers to innovative statistical tools that enhanced existing research models. These tools not only assessed the predictability of empirical phenomena but also quantitatively measured the latent trait of interest, namely potential intelligibility, facilitating the comparison of research hypotheses related to this trait. However, the presented tools introduce new challenges for researchers seeking their implementation. These challenges emerge from two distinct aspects: one methodological and the other practical. In the methodological domain, researchers need familiarity with Bayesian methods and the principled formulation of assumptions regarding the data-generating process and research inquiries. This entails understanding and addressing each of the data and research challenges within the context of a statistical (probabilistic) models. Conversely, in the practical domain, researchers need familiarity with probabilistic programming languages (PPLs), which are designed for specifying and obtaining inferences from probabilistic models – the core of Bayesian methods. To ensure the successful utilization of this new statistical tool, this study addressed both challenges by providing comprehensive, step-by-step guidance in the form of a digital walk-through document (see Sect. Open Science Statement).

### Limitations and further research

This study provided valuable insights into the use of a novel approach to simultaneously address the different data features of entropy scores in speech intelligibility research. However, it is important to acknowledge the limitations of this study and explore potential avenues for future research. Firstly, the study interpreted potential intelligibility as an unobserved latent trait of speakers influencing the likelihood of observing a set of entropy scores. These scores, in turn, reflected the transcribers’ ability to decode words in sentences produced by the speakers. Despite this practical approach, the construct validity of the latent trait heavily depended on the listeners’ appropriate understanding and execution of the transcription task. Construct validity, as defined by Cronbach and Meehl (1955), refers to the extent to which a set of manifest variables accurately represents a concept that cannot be directly measured. Considering the study assumed the transcription task set by Boonen et al. (2023) was properly understood and executed, it expected that the potential intelligibility reflected the overall speech intelligibility of speakers. However, the study did not delve into the general epistemological considerations regarding the connection between the latent variable and the concept.

Secondly, the study revealed a notable lack of unequivocal support for one of the models among the compared set. This outcome may be attributed to factors such as the insufficient sample size of speakers, the inadequate representation of the populations of speakers (referred to as selection bias), and the imprecise measurement of the latent variable. Small sample sizes and selection bias yield data with limited outcome and covariates ranges, leading to biased and imprecise parameter estimates (Everitt & Skrondal, 2010). Moreover, fueled by the reduced measurement precision, these issues can result in models with diminished statistical power and a higher risk of type I or type II errors (McElreath, 2020). Consequently, future research should consider extending this study by conducting formal sample size planning. This entails assessing the impact of expanding the speakers’ pool on testing research hypotheses or increasing the number of speech samples, transcriptions, and listeners to enhance the precision of the potential intelligibility estimates. With these insights, future investigations could contemplate increasing the speaker sample with a group that adequately represents the population of interest. However, this must be done while mindful of the pragmatic limitations associated with transcription tasks, specifically considering the costs and time-intensiveness of the procedure.

Thirdly, the study presented an illustrative example for the investigation of research hypotheses within the model’s framework. However, it did not offer an exhaustive evaluation of all factors influencing intelligibility, which are thoroughly explored in the works of Niparko et al. (2010), Boons et al. (2012), Gillis (2018), and Fagan et al. (2020). Consequently, the study could not discard the presence of unobservable variables that might bias the parameter estimates, potentially impacting the inferences provided. Hence, future research should consider integrating appropriate causal hypotheses about these factors into the proposed models, as proper covariate adjustment facilitates the production of unbiased and precise parameter estimates (Cinelli et al., 2022; Deffner et al., 2022).

Lastly, this study proposes two directions for future exploration in speech intelligibility research. Firstly, there is an opportunity to investigate alternative methods for assessing speech intelligibility beyond transcription tasks and entropy scores. The experimental design of transcription tasks imply that the procedure may be time-intensive and costly. Thus, exploring less time-intensive or more cost-effective procedures, that still offer comparable precision in intelligibility estimates, could benefit both researchers and speech therapists alike. One example of such a method is comparative judgment (CJ), where judges compare and score the perceived intensity of a trait between two stimuli (Thurstone, 1927). CJ has gained increasing attention in educational assessment, with several studies demonstrating its validity in assessing various tasks within student work, as shown in Pollitt (2012a), Pollitt (2012b), Lesterhuis (2018), van Daal (2020), and Verhavert et al. (2019). The work of Boonen et al. (2020) illustrates the potential of this methodology to assess intelligibility. In their study, the authors assessed the overall speech quality of hearing-impaired children using pairwise comparisons of uttered speech samples, while scoring the results in a dichotomous manner. Nevertheless, there is significant room for extending their application. For instance, researchers can perform retrospective power analysis to ascertain the power of the study’s claims (see Kruschke, 2015, 393–94). Furthermore, the application can be extended to other unexplored variants of the CJ method, such as ordered CJ (Pritikin, 2020) or multidimensional dichotomous CJ.

Conversely, a second avenue for exploration involves integrating diverse data types and evaluation methods to assess individuals’ intelligibility. This can be accomplished by leveraging two features of Bayesian methods: their flexibility and the concept of Bayesian updating. Bayesian methods possess the flexibility to simultaneously handle various data types. Additionally, through Bayesian updating, researchers can integrate information from the posterior distribution of parameters as priors in models for subsequent evaluations. Ultimately, this could enable researchers to assess speakers’ intelligibility progress without committing to a specific data type or evaluation method. This advancement could mirror the emergence of second-generation Structural Equation Models proposed by Muthén (2001), where models facilitate the combined estimation of categorical and continuous latent variables. However, in the context of future research, the proposal would facilitate the estimation of latent variables using a combination of data types and evaluation methods, contingent upon the fulfillment of construct validity by those evaluation methods.

## Conclusion

This study have highlighted the effectiveness of the Bayesian beta-proportion GLLAMM to collectively address several key data features when investigating unobservable and complex traits. The study used speech intelligibility and entropy scores as a motivating example. The results have demonstrated that the proposed model consistently outperforms the normal LMM in predicting the empirical phenomena. Moreover, the model exhibits the ability to quantify the latent potential intelligibility of speakers, allowing for the ranking and comparison of individuals based on the latent trait while accommodating associated uncertainties. Additionally, the proposed model have facilitated the exploration of research hypotheses concerning the influence of speaker-related factors on potential intelligibility, where the integration and comparison of these hypotheses within the model’s framework was a straightforward task.

However, the introduction of these innovative statistical tools presents new challenges for researchers seeking implementation. These challenges encompass the principled formulation of assumptions about the data-generating processes and research inquiries, along with the need for familiarity with probabilistic programming languages (PPLs) essential for implementing Bayesian methods. Nevertheless, the study suggests several promising avenues for future research, including causal hypothesis formulation, and the exploration and integration of novel evaluation methods for assessing intelligibility. The insights derived from this study hold implications for both researchers and data analysts interested in quantitatively measuring intricate, unobservable constructs, while predicting accurately the empirical phenomena.

## Data Availability

The data is delivered upon request.

## Appendix

### Entropy scores calculation

This section exemplified the entropy calculation procedure. For that purpose, the words in position two, three, four and five observed in Table 1 were used. These words were assumed present in the first sentence, produced by the first speaker assigned to the first block, and transcribed by five listeners ($w=\{\text{2,3},\text{4,5}\}$, $s=1$, $i=1$, $b=1$, $J=5$). For second word, the first four listeners identified the word type *jongen* $\left({T}_{j1}\right)$, while the last identified the word type *hond* $\left({T}_{j2}\right)$. Therefore, two word types were identified ($K=2$), with proportions equal to $\{{p}_{1},{p}_{2}\}=$ $\{4/5,1/5\}=$ $\{\text{0.8,0.2}\}$, with an entropy score equal to:

$${H}_{2111}=\frac{-\left[0.8\cdot lo{g}_{2}\left(0.8\right)+0.2\cdot lo{g}_{2}\left(0.2\right)\right]}{lo{g}_{2}\left(5\right)}\approx 0.3109$$

For the fourth word, two listeners identified the word type *een* $\left({T}_{j1}\right)$, one listener the word type *de* $\left({T}_{j2}\right)$, and another the word *geen* $\left({T}_{j3}\right)$. In addition, a blank space *[B]* is a symbol that defined the absence of a word in a space where a word was expected during the alignment procedure, as compared with other transcriptions. Notice that for calculation purposes, because the blank space was not expected in such position, it was considered as a different word type. Consequently four word types were registered ($K=4$), with proportions equal to $\{{p}_{1},{p}_{2},{p}_{3},{p}_{4}\}=$ $\{2/5,1/5,1/5,1/5\}=$ $\{\text{0.4,0.2,0.2,0.2}\}$ with an entropy score equal to:

$${H}_{4111}=\frac{-\left[0.4\cdot lo{g}_{2}\left(0.4\right)+3\cdot 0.2\cdot lo{g}_{2}\left(0.2\right)\right]}{lo{g}_{2}\left(5\right)}\approx 0.8277$$

For the fifth word, each listener transcribed a different word. It is important to highlight that when a listener did not identify a complete word, or part of it, (s)he was instructed to write *[X]* in that position. However, for the calculation of the entropy score, if more than one listener marked an unidentifiable word with *[X]*, each one of them was considered a different word type. This was done to avoid the artificial reduction of the entropy score, as *[X]* values already indicated the word’s lack of intelligibility. Consequently, five word types were observed, ${T}_{j1}=$ *kikker*, ${T}_{j2}=$*[X]*, ${T}_{j3}=$ *kokkin*, ${T}_{j4}=$ *kikkers*, ${T}_{j5}=$*[X]* ($K=5$), with proportions equal to $\{{p}_{1},{p}_{2},{p}_{3},{p}_{4},{p}_{5}\}=$ $\{1/5,1/5,1/5,1/5,1/5\}=$ $\{\text{0.2,0.2,0}.\text{2,0.2,0.2}\}$, with an entropy score equal to:

$${H}_{5111}=\frac{-\left[5\cdot 0.2\cdot lo{g}_{2}\left(0.2\right)\right]}{lo{g}_{2}\left(5\right)}=1$$

Lastly, for the third word, the first two listeners identified the word type *ziet* $\left({T}_{j1}\right)$, the next two listeners identified the word type *zag* $\left({T}_{j2}\right)$, while the last one identified the word type *zoekt* $\left({T}_{j3}\right)$. Consequently, three word types were identified ($K=3$), with proportions equal to $\{{p}_{1},{p}_{2},{p}_{3}\}=$ $\{2/5,2/5,1/5\}=$ $\left\{\text{0.4,0.4,0.2}\right\}$, with an entropy score equal to:

$${H}_{2111}=\frac{-\left[2\cdot 0.4\cdot lo{g}_{2}\left(0.4\right)+0.2\cdot lo{g}_{2}\left(0.2\right)\right]}{lo{g}_{2}\left(5\right)}\approx 0.6555$$

Importantly, the last example showcased the major difference between entropy and measures of accuracy based on the percentage of (un)intelligible words. Entropy scores employ all word type proportions in their calculations, effectively capturing the agreement and disagreement among listeners’ word transcriptions (Boonen et al., 2023). In contrast, the percentage of (un)intelligible words discards most word type proportions in favor of *simpler* agreement or disagreement percentages. For example, an agreement percentage could be reflected by the proportion of the most frequent word, i.e., $\text{max}\left\{\text{0.4,0.4,0.2}\right\}=0.4$, or by other similar percentages detailed in the works of Flipsen (2006) and Lagerberg et al. (2014).

**Table 6:** WAIC comparison for selected models. **Note:** The table is sorted based on weight from most to least plausible model(s) for the data

Model	DIC	WAIC	SE	dWAIC	dSE	pWAIC	Weight
10	– 9741.66	– 9630.63	276.64	0.00		55.52	1.00
7	– 9649.54	– 9586.00	274.50	44.63	17.89	31.77	0.00
4	– 2670.62	– 2024.84	127.02	7605.78	263.22	322.89	0.00
1	– 2278.68	– 1761.10	101.80	7869.53	266.54	258.79	0.00

**Table 7:** PSIS comparison for selected models. **Note:** The table is sorted based on weight from most to least plausible model(s) for the data

Model	DIC	PSIS	SE	dPSIS	dSE	pPSIS	Weight
10	– 9741.66	– 9629.27	276.74	0.00		56.19	1.00
7	– 9649.54	– 9585.92	274.56	43.36	17.67	31.81	0.00
4	– 2670.62	– 2007.66	128.57	7621.61	263.60	331.48	0.00
1	– 2278.68	– 1753.71	102.09	7875.57	266.54	262.48	0.00

**Table 8:** WAIC comparison for all models. **Note:** The table is sorted based on weight from most to least plausible model(s) for the data

Model	DIC	WAIC	SE	dWAIC	dSE	pWAIC	Weight
11	– 9741.51	– 9632.24	276.80	0.00		54.63	0.46
12	– 9741.49	– 9631.66	276.82	0.58	1.00	54.91	0.34
10	– 9741.66	– 9630.63	276.64	1.61	2.97	55.52	0.20
9	– 9649.15	– 9586.67	274.35	45.56	18.01	31.24	0.00
8	– 9649.05	– 9586.41	274.33	45.83	18.01	31.32	0.00
7	– 9649.54	– 9586.00	274.50	46.24	18.19	31.77	0.00
6	– 2669.28	– 2027.11	126.86	7605.13	263.15	321.08	0.00
4	– 2670.62	– 2024.84	127.02	7607.40	263.22	322.89	0.00
5	– 2669.28	– 2024.58	127.06	7607.66	263.24	322.35	0.00
3	– 2279.58	– 1762.08	101.79	7870.16	266.68	258.75	0.00
1	– 2278.68	– 1761.10	101.80	7871.14	266.64	258.79	0.00
2	– 2279.35	– 1760.36	101.86	7871.88	266.69	259.49	0.00

**Table 9:** PSIS comparison for all models. **Note:** The table is sorted based on weight from most to least plausible model(s) for the data

Model	DIC	PSIS	SE	dPSIS	dSE	pPSIS	Weight
11	– 9741.51	– 9631.16	276.88	0.00		55.17	0.46
12	– 9741.49	– 9630.70	276.90	0.47	1.01	55.39	0.36
10	– 9741.66	– 9629.27	276.74	1.89	2.84	56.19	0.18
9	– 9649.15	– 9586.58	274.41	44.58	17.91	31.28	0.00
8	– 9649.05	– 9586.33	274.39	44.83	17.91	31.36	0.00
7	– 9649.54	– 9585.92	274.56	45.24	18.10	31.81	0.00
6	– 2669.28	– 2009.22	128.46	7621.94	263.52	330.03	0.00
4	– 2670.62	– 2007.66	128.57	7623.50	263.60	331.48	0.00
5	– 2669.28	– 2006.49	128.71	7624.67	263.62	331.39	0.00
3	– 2279.58	– 1754.43	102.07	7876.73	266.68	262.57	0.00
1	– 2278.68	– 1753.71	102.09	7877.46	266.64	262.48	0.00
2	– 2279.35	– 1752.86	102.13	7878.30	266.68	263.24	0.00

## Code availability

All the code utilized in this research is available in the different notebooks and CODE LINKS referenced in the digital document. The digital document is located at: https://jriveraespejo.github.io/paper1_manuscript/.
